# Immunophenotypic characterization of CSF B cells in virus-associated neuroinflammatory diseases

**DOI:** 10.1371/journal.ppat.1007042

**Published:** 2018-04-30

**Authors:** Yoshimi Enose-Akahata, Shila Azodi, Bryan R. Smith, Bridgette Jeanne Billioux, Ashley Vellucci, Nyater Ngouth, Yuetsu Tanaka, Joan Ohayon, Irene Cortese, Avindra Nath, Steven Jacobson

**Affiliations:** 1 Viral Immunology Section, National Institute of Neurological Disorders and Stroke, National Institutes of Health, Bethesda, MD, United States of America; 2 Section of Infections of the Nervous System, National Institute of Neurological Disorders and Stroke, National Institutes of Health, Bethesda, MD, United States of America; 3 Department of Immunology, Graduate School and Faculty of Medicine, University of the Ryukyus, Okinawa, Japan; 4 Neuroimmunology Clinic, National Institute of Neurological Disorders and Stroke, National Institutes of Health, Bethesda, MD, United States of America; Imperial College London, UNITED KINGDOM

## Abstract

Intrathecal antibody synthesis is a well-documented phenomenon in infectious neurological diseases as well as in demyelinating diseases, but little is known about the role of B cells in the central nervous systems. We examined B cell and T cell immunophenotypes in CSF of patients with HTLV-1-associated myelopathy/tropical spastic paraparesis (HAM/TSP) compared to healthy normal donors and subjects with the other chronic virus infection and/or neuroinflammatory diseases including HIV infection, multiple sclerosis (MS) and progressive multifocal leukoencephalopathy. Antibody secreting B cells (ASCs) were elevated in HAM/TSP patients, which was significantly correlated with intrathecal HTLV-1-specific antibody responses. High frequency of ASCs was also detected in patients with relapsing-remitting multiple sclerosis (RRMS). While RRMS patients showed significant correlations between ASCs and memory follicular helper CD4^+^ T cells, CD4^+^CD25^+^ T cells were elevated in HAM/TSP patients, which were significantly correlated with ASCs and HTLV-1 proviral load. These results highlight the importance of the B cell compartment and the associated inflammatory milieu in HAM/TSP patients where virus-specific antibody production may be required to control viral persistence and/or may be associated with disease development.

## Author summary

Regulation of the local immune response is crucial in protecting the central nervous system (CNS) from viral infection and immunopathologically mediated tissue damage. Intrathecal antibody synthesis is a well-documented phenomenon in infectious and demyelinating neurological diseases, but little is known about the CNS microenvironment related to this increased humoral immune response in disease and healthy controls. Comparison of CSF immune phenotyping highlights that B cell/T cell interactions may be involved in the development and maturation of B cells in the CNS of virus-associated neuroinflammatory diseases. Characterization of CSF immune responses that are associated with a neuroinflammatory milieu may provide evidence for a pathogenic “signature” of an immunopathogenic process in virus-associated neurologic diseases.

## Introduction

Various inflammatory neurologic diseases are associated with viral infections. These agents may cause direct cellular damage of infected cells associated with immunological alterations such as chronic activation, immunodeficiency and infiltration of inflammatory cells into the central nervous system (CNS) that underlie the pathogenesis of inflammatory neurologic disorders. Intrathecal antibody synthesis is a well-documented phenomenon in infectious and demyelinating neurologic diseases. Various viral infections of the CNS including polio, rabies, mumps, herpes simplex virus and Japanese encephalitis virus are characterized by intrathecal antibody production in cerebrospinal fluid (CSF) and/or presence of local antibody-secreting B cells (ASCs) [1, 2]. While virus-specific antibodies play an important role in the control of viral infections in the CNS, intrathecal antibody synthesis has been associated with both protective and pathogenic functions in chronic infection and immune-mediated disorders of the CNS.

Human T cell lymphotropic virus 1 (HTLV-1) is a human retrovirus that infects over 20 million people worldwide. Only a small proportion of infected people develop either adult T cell leukemia/lymphoma (ATL) [3] or HTLV-1-associated myelopathy/tropical spastic paraparesis (HAM/TSP) [4, 5]. HAM/TSP is a chronic, progressive neurological disease characterized by perivascular inflammatory infiltrates in the brain and spinal cord [6]. High frequencies of effector T cells have been demonstrated in peripheral blood with even higher frequencies in CSF of patients with HAM/TSP [7–9]. As definitive laboratory diagnosis of HAM/TSP is based on the presence of anti-HTLV-1 antibodies in the blood and CSF, robust humoral immune responses against HTLV-1 antigens have been reported [5, 10, 11]. Thus, chronically activated immune responses and infiltration of inflammatory cells into the CNS have been suggested to underlie the pathogenesis of HAM/TSP. Intrathecal antibody synthesis against HTLV-1 has been also reported, as evidenced by the presence of HTLV-1-specific antibodies and oligoclonal IgG bands (OCB) in CSF of HAM/TSP patients [12–15]. Intrathecal antibody response to HTLV-1 inversely correlates with higher HTLV-1 proviral loads (PVL) and a worse prognostic outcome [16]. Moreover, antibodies against two HTLV-1 viral products, Tax and Gag p24, have been reported to cross-react with host antigens, heterogeneous ribonucleoprotein A1 (hnRNP A1) and peroxiredoxin-1 (PrX-1), respectively, suggesting that molecular mimicry may play a role in the pathogenesis of HAM/TSP [17, 18]. Since little is known about the role of B cells in the CNS of HAM/TSP patients, it is of interest to characterize and compare local B cell immune responses associated with the inflammatory milieu in the other chronic virus infection or neuroinflammatory diseases, such as multiple sclerosis (MS) which has clinical features that resemble HAM/TSP [19].

MS is a chronic, neurodegenerative inflammatory disease of the CNS, which leads to demyelination and progressive neurological disability. Based on the disease course, there are three main forms of MS. The more common course, relapsing-remitting MS (RRMS) is characterized by clinical episodes interspersed by periods of stability, affects twice as many women than men and in 40% of patients later develops a secondary progressive MS (SPMS) within ten years. Approximately 10% of patients experience a primary progressive MS (PPMS), which is characterized by gradual neurological dysfunction with or without exacerbations [20]. Although the etiology of MS is still unknown, viruses, such as Epstein-Bar virus (EBV) and Human herpes virus type 6 (HHV-6), are considered to be leading candidates associated with the pathogenesis of MS [21]. A hallmark of MS is the detection of OCB in the CSF that are associated with long term B cell survival in this compartment. Interestingly, recent studies have also demonstrated that in part, these CSF OCB in MS are specific for infectious pathogens and host antigens [22]. Currently, B cell depletion, with medications such as rituximab and ocrelizumab, is a promising MS treatment strategy [23]. However, such therapies have been also linked to progressive multifocal leukoencephalopathy (PML) which is a rare, often fatal, demyelinating disease caused by reactivation of the ubiquitous JC virus [24]. These observations suggest that CSF B cells associated with antibody synthesis may function to control viral replication in the CNS. It is therefore important to define the CNS microenvironment involved in recruitment and retention of B cell and ASCs in patients with virus-associated neuroinflammatory diseases.

Antigen-specific antibodies in CSF are either derived from the blood (leakage through the blood-brain-barrier) or are synthesized locally within the CNS. In patients with MS, there is a persistence of clonally expanded B cells and non-dividing plasma cells in the CSF, as well as increase of chemokines and cytokines involved in B cell migration, differentiation, and long-term survival in the CNS [25–27]. These results suggest that the presence of ASCs in the CNS and the associated environment are critical aspects of the immune response. Interaction between CXCL13 (B cell-attracting chemokine-1) and its receptor CXCR5 is responsible for the migration of B cells and a subset of T cells in the follicular areas of lymphoid tissues called follicular helper CD4^+^ T cells (Tfh cells) [28–31]. Tfh cells are generally characterized by their expression of the chemokine receptor CXCR5, the transcription factor BCL6, and the inhibitory molecule PD-1, expression of high levels of IL-21, and their promotion of B cell help [32]. Recently, it has been shown that after specific activation, human blood CXCR5^+^CD4^+^ T cells might correspond to a circulating pool of memory Tfh cells [33]. This pool is distinct from the Th1, Th2, and Th17 subsets and can prompt naive and memory B cells to differentiate into ASCs, mainly through IL-21- and ICOS-induced signals [33, 34]. Although it remains unclear how circulating memory Tfh cells relate to tissue Tfh cells, recent studies suggested that a subset of blood-circulating memory CXCR5^+^CD4^+^ T cells that are characterized by stable and moderate expression of the Tfh cell marker PD-1 most resemble tissue Tfh cells among resting memory CD4^+^ T cells in terms of B cell help functionality and transcriptional signature [33, 35]. It has been reported that IL-21 mRNA was elevated in peripheral blood CD4^+^ T cells of MS patients and IL-21 expressing CD4^+^ T cells were detected in MS lesions [36, 37]. In addition, increased circulating memory Tfh cells and plasma IL-21 level as well as CSF IL-21 level have been reported to be significantly elevated in MS patients than in controls with non-inflammatory neuronal diseases [38]. These studies suggested that memory Tfh cells may be involved in B cell help through IL-21. IL-21 is a member of the common γ chain family of cytokine that also includes IL-2, IL-7, IL-9 and IL-15, and promotes B-cell growth, differentiation, and class-switching [39]. Viral genes (such as HTLV-1 Tax) have been shown to transactivate some common γ chain family of cytokines including IL-21 and its receptor (IL-21R) in human T cells [40–42], and therefore it is of interest to understand the molecular cues of T cell/B cell interaction in the CNS microenvironment in patients with viral mediated neuroinflammatory disease.

In this study, we analyzed B cell and T cell immunophenotypes in CSF of subjects with the chronic virus infection and/or neuroinflammatory diseases including HAM/TSP patients, HTLV-1-infected asymptomatic carriers (ACs), HIV-infected subjects treated with antiretroviral drugs, MS patients (RRMS and PPMS) and PML patients, compared to healthy normal donors (NDs). Comparison of CSF B cell subsets revealed that ASCs are increased in the CSF of all or a subset of these patients suggesting that B cell-mediated immune activation might be a critical aspect of the regulation and/or the pathogenesis of neuroinflammatory diseases associated with (or suspected of being associated with) viruses. Moreover, we demonstrate that increased ASCs are correlated with CD4^+^CD25^+^ T cells in the CSF of HAM/TSP patients whereas it is correlated with memory Tfh cells in MS patients. These results highlight the importance of the B cell compartment and the associated inflammatory milieu where production of antigen-specific antibody may be required to control viral persistence and/or may be associated with disease development in neuroinflammatory diseases.

## Results

### Detection of B cells in CSF of patients with neuroinflammatory diseases

**Fig 1:**
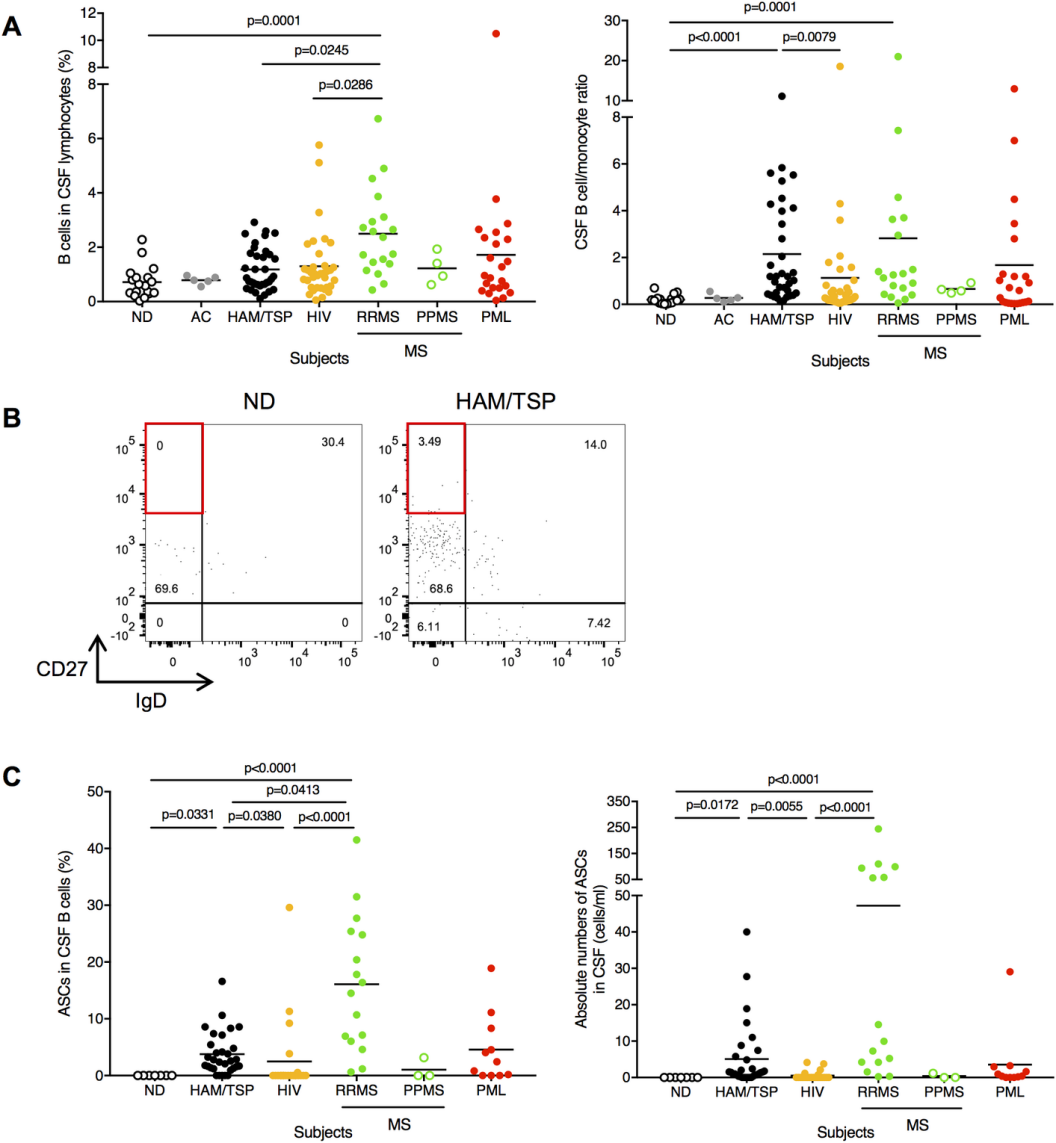
Detection of B cells in CSF of chronic virus infection and/or neuroinflammatory diseases. (A) Comparison of frequencies of B cells (left) and B cell/monocyte ratio (right) in CSF using Kruskal-Wallis test with Dunn’s test. The horizontal line represents the mean. (B) Detection of ASCs subset in B cells of CSF. (Left) Representative dot plots of IgD and CD27 staining in CSF CD19^+^ B cells of a ND and a HAM/TSP patient. IgD^-^ CD27^++^ subsets (red rectangles) represent ASCs. (C) Comparison of frequencies of ASCs (left) and absolute number of ASCs (right) in CSF using Kruskal-Wallis test with Dunn’s test. The horizontal line represents the mean.

In situ histopathological studies in spinal cord of HAM/TSP patients demonstrated that T cells including both CD4^+^ and CD8^+^ T cells were detected depending on the duration of illness whereas B cells were only rarely observed [6]. However, since elevated intrathecal antibody synthesis have been demonstrated in HAM/TSP patients, we hypothesized that B cell recruitment and/or differentiation may also be present in CSF of patients. To confirm the presence of B cells in the CSF of subjects with chronic virus infection and/or neuroinflammatory diseases, we examined a large collection of CSF lymphocytes obtained from HAM/TSP patients, ACs and the other chronic virus infection and/or neuroinflammatory diseases including HIV-infected subjects, MS patients and PML patients (Table 1). We also had the unique opportunity to collect CSF from eighteen NDs as controls (Table 1). T cells were the predominant population in CSF lymphocytes of NDs (about 60 to 80% of lymphocytes) and CD4^+^ T cells are more prevalent than CD8^+^ T cells in our study (the average of CD4/CD8 ratio; 3.9). Low levels of B cells were detected in lymphocytes from ND CSF, but were significantly elevated in patients with RRMS (Fig 1A, left graph). B cell/monocyte ratio which has been previously shown to be an indicator of rapid progression in MS patients [43] was higher in CSF of HAM/TSP patients as well as RRMS patients compared to NDs (Fig 1A, right graph). Importantly, B cell frequency and B cell/monocyte ratio were low in the CSF of ACs, which was comparable to those in NDs (Fig 1A). Flow cytometric analysis was able to differentiate B cell subpopulation into five subsets, including naïve (IgD^+^CD27^-^), unswitched memory (IgD^+^CD27^+^), double negative (IgD^-^CD27^-^), switched memory (IgD^-^CD27^+^) B cells and ASCs (IgD^-^CD27^++^) in CSF of a ND and a HAM/TSP patient (Fig 1B). Representative dot plots demonstrate that switched memory B cells were predominantly detected in the CSF of both subjects (Fig 1B). Interestingly, ASCs were elevated in the CSF of HAM/TSP patient compared to a ND (red rectangles in Fig 1B). ASCs were not detected in the CSF of NDs, but the presence of elevated ASCs in CSF was also higher in patients with RRMS, HAM/TSP and PML (Table 2). Group analysis of B cell subset also revealed that high frequency and absolute number of ASCs was detected in the CSF of HAM/TSP patients as well as RRMS patients (Fig 1C). ASCs were not or rarely detected in the CSF of subjects without neurologic diseases including ACs and HIV-infected subjects, although B cell phenotyping was able to be analyzed in only two ACs due to limited CSF cell number (Table 2). It is also of interest that there was undetectable or a low frequency of ASCs in the CSF of PPMS patients although only a small number of subjects was analyzed (Fig 1C). These results demonstrated that B cell recruitment and/or differentiation may be present in CSF of subjects with chronic virus infection and/or neuroinflammatory diseases.

**Table 1:** Characteristics of subjects for flow cytometric analysis.

Group	Sample	Age	Gender	CSF cell concentration (cells/μl)
	Blood/CSF, n	Mean ± SD	Female n (%)	Mean ± SD
ND	31 / 18	47.26 ± 9.64	15 (48.39%)	1.25 ± 0.61
AC	8 / 6	49.75 ± 11.96	6 (75.00%)	2.86 ± 2.51
HAM/TSP	39 / 36	57.67 ± 11.07	31 (79.45%)	10.00 ± 12.30
HIV	38 / 38	53.03 ± 6.318	16 (42.11%)	1.90 ± 1.69
MS (RRMS)	22 / 19	39.59 ± 12.88	15 (68.18%)	7.93 ± 10.83
(PPMS)	4 / 4	52.25 ± 8.958	1 (25.00%)	1.71 ± 1.60
PML	28 / 28	54.79 ± 11.95	11 (39.29%)	2.87 ± 4.34

**Table 2:** Detection of antibody secreting B cells in CSF.

Group	Total subjects in B cell analysis, n	Subjects with ASCs, n (%)	Fisher's exact test*
ND	7	0 (0.00%)	
AC	2	0 (0.00%)	ns
HAM/TSP	31	26 (83.87%)	p<0.0001
HIV	22	5 (22.73%)	ns
MS (RRMS)	16	16 (100.00%)	p<0.0001
(PPMS)	3	1 (33.33%)	ns
PML	11	8 (72.73%)	p = 0.0040

* ns; not significant

### Correlation of ASCs with intrathecal HTLV-1-specific antibody responses in HAM/TSP patients

**Fig 2:**
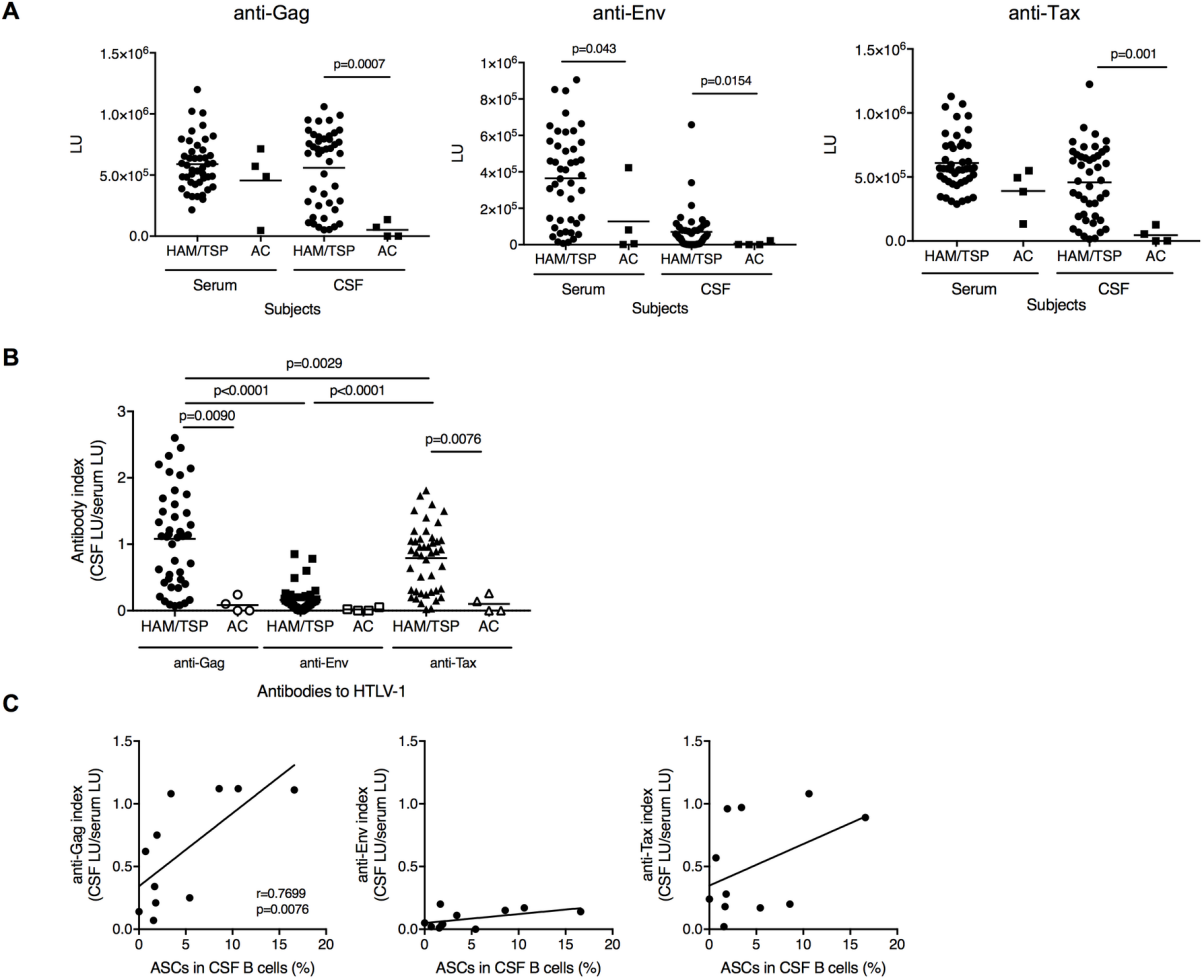
Antibody responses against HTLV-1 in CSF of HTLV-1-infected subjects. (A) Comparison of antibody responses against HTLV-1 Gag, Env and Tax in serum and CSF of ACs and HAM/TSP patients using Mann-Whitney Test. (B) Comparison of CSF/serum anti-HTLV-1 Gag, Env and Tax antibody ratio of HAM/TSP patients and ACs using Paired T test or Mann-Whitney Test. All the data were obtained from HAM/TSP patients (n = 44) and ACs (n = 4). The horizontal line represents the mean. (C) Correlation of ASCs in CSF B cells with anti-Gag, anti-Env and anti-Tax antibody index in HAM/TSP patients (n = 11) using Spearman’s rank correlation test.

Increased ASCs may be involved in intrathecal antibody synthesis in CSF of subjects with chronic virus infection and/or neuroinflammatory diseases. Since HAM/TSP patients had higher antibody responses for HTLV-1 virus proteins, Gag, Env and Tax, in serum [11], we next analyzed antibody responses for HTLV-1 antigens in CSF and serum of HAM/TSP patients and ACs to examine whether HTLV-1-specific antibody synthesis is associated with ASC in CSF of HAM/TSP patients. Robust antibody responses for Gag, Env and Tax were observed in CSF of HAM/TSP patients compared to ACs (Fig 2A). Immunoreactivities against Gag and Tax were detected in the CSF of all HAM/TSP patients and immunoreactivity against Env was detected in 93.2% of the CSF. In the CSF, the mean anti-Gag level was elevated comparable to that in the serum, but other anti-HTLV-1 antibodies, anti-Env and anti-Tax antibody level, were significantly lower in the CSF than in serum (p<0.0001 and p = 0.0027, respectively; Fig 2A). When the data were analyzed as an index of CSF immunoreactivity to serum immunoreactivity against each HTLV-1 antigen, the anti-Gag antibody index was higher than anti-Tax antibody index and significantly more elevated than anti-Env antibody index in HAM/TSP patients (Fig 2B). In contrast, ACs showed low levels of CSF/serum antibody indexes to all three antigens (Fig 2B). These results demonstrate that intrathecal anti-Gag and anti-Tax antibody synthesis are significantly elevated in HAM/TSP patients compared to those in ACs. Moreover, increased ASCs in CSF of HAM/TSP patients were significantly correlated with anti-Gag antibody index, but not with anti-Env or anti-Tax antibody indexes (Fig 2C). These results suggested that ASCs were involved in the intrathecal antibody synthesis, especially anti-HTLV-1 Gag, in the CSF of HAM/TSP patients.

### Detection of memory Tfh cells in CSF

**Fig 3:**
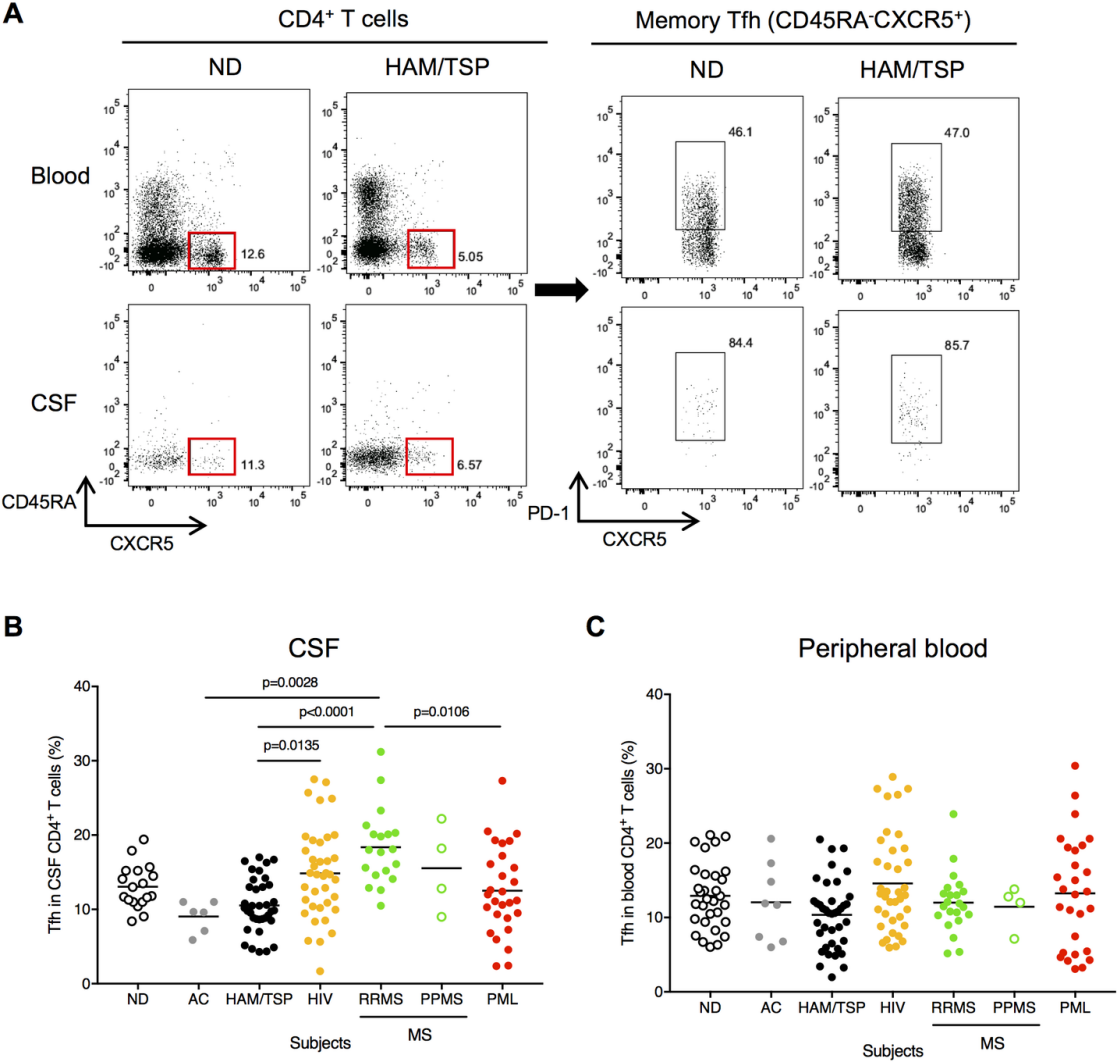
Detection of memory Tfh cells in CSF of chronic virus infection and/or neuroinflammatory diseases. (A) Detection of memory Tfh cells in peripheral blood and CSF. Representative dot plots of memory Tfh cells (CXCR5^+^ CD45RA^-^; red rectangles) in CD4^+^ T cells and PD-1 expression in memory Tfh cells of peripheral blood and CSF of a ND and a HAM/TSP patient. (B) Comparison of frequency of memory Tfh cells in CSF using Kruskal-Wallis test with Dunn’s test. The horizontal line represents the mean. (C) Comparison of frequency of memory Tfh cells in peripheral blood using Kruskal-Wallis test with Dunn’s test. The horizontal line represents the mean.

In the CSF of MS patients, it is well documented that clonally expanded B cells and non-dividing plasma cells persist with increased levels of chemokines and cytokines associated with B cell migration and differentiation [22], suggesting that CXCR5^+^ Tfh cells can also migrate and interact with B cells in the CNS. As memory Tfh cells have been reported to promote B cell growth, differentiation and class switching and to resemble tissue Tfh cells [32, 33, 35], we examined whether CXCR5-expressing memory Tfh cells are present in the CSF and peripheral blood of HAM/TSP patients compared to NDs, ACs and patients with the other chronic virus infection and/or neuroinflammatory diseases. Fig 3A shows representative results of memory Tfh cells (CXCR5^+^ CD45RA^-^; red rectangles) in CD4^+^ T cells of peripheral blood and CSF of a ND and a HAM/TSP patient. As Tfh cells are also characterized by the expression of the inhibitory molecule PD-1 [32], high frequency of PD-1 was detected in CSF, much higher than in peripheral blood, in memory Tfh cells of both ND and HAM/TSP patient (Fig 3A). Memory Tfh cells were detected in both CSF and peripheral blood of all the subjects (Fig 3B and 3C). Comparison of memory Tfh cells in CD4^+^ T cells demonstrated that the frequency of memory Tfh cell subset was slightly decreased in CSF of HAM/TSP patients compared to those in NDs while RRMS patients showed an increase of memory Tfh cells in the CSF (Fig 3B). In subjects with HIV, PPMS and PML patients, the frequency of memory Tfh cells varied in the CSF (Fig 3B). Intriguingly, memory Tfh cells were also slightly decreased in the CSF of ACs compared to that in NDs (Fig 3B). In CD4^+^ T cells of peripheral blood, there was no significant differences of memory Tfh cell frequencies in all the groups (Fig 3C). Dynamic changes of memory Tfh cells were detected in CSF of each group despite the increased levels of ASCs detected in the CSF of each of these cohorts of virus-associated neurologic diseases, suggesting that CSF B cells might be regulated by a different mechanism in each chronic virus infection and/or neuroinflammatory disorder.

### Detection of CD4^+^CD25^+^ T cells in CSF

**Fig 4:**
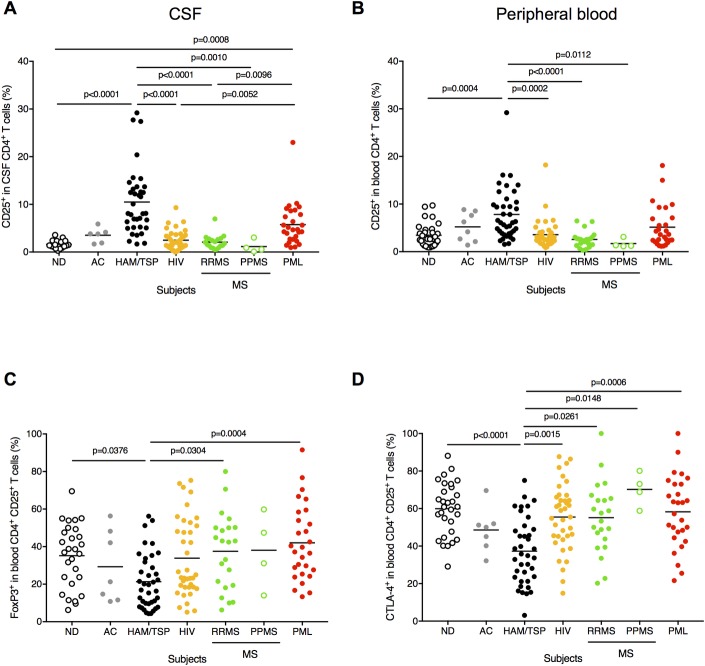
Detection of CD4^+^CD25^+^ T cells in CSF of chronic virus infection and/or neuroinflammatory diseases. (A) Comparison of frequency of CD4^+^CD25^+^ T cells in CSF using Kruskal-Wallis test with Dunn’s test. The horizontal line represents the mean. (B) Comparison of frequency of CD4^+^CD25^+^ T cells in peripheral blood using Kruskal-Wallis test with Dunn’s test. The horizontal line represents the mean. (C) Comparison of frequency of FoxP3 in peripheral blood CD4^+^CD25^+^ T cells using Kruskal-Wallis test with Dunn’s test. The horizontal line represents the mean. (D) Comparison of frequency of CTLA-4 in peripheral blood CD4^+^CD25^+^ T cells using Kruskal-Wallis test with Dunn’s test. The horizontal line represents the mean.

In HAM/TSP patients, CD4^+^CD25^+^ T cells are the predominant reservoir for HTLV-1 and induce various cytokines including IFN-γ [44]. Higher HTLV-1 PVL was detected in CSF compared to PBMC in HAM/TSP patients [45, 46], suggesting that HTLV-1-infected CD4^+^ T cells can be recruited into the CNS and may alter the inflammatory milieu in the CNS of HTLV-1-infected subjects. We next examined whether CD4^+^CD25^+^ T cells are present in the CSF and peripheral blood of HAM/TSP patients compared to that of NDs, ACs and subjects with chronic virus infection and/or neuroinflammatory diseases. The frequency of CD4^+^CD25^+^ T cells were significantly higher in both CSF and peripheral blood of HAM/TSP patients compared to NDs and subjects with HIV and MS (Fig 4A and 4B, respectively). CD4^+^CD25^+^ T cells were also significantly higher in the CSF of patients with PML compared to NDs, but not in the peripheral blood (Fig 4A and 4B).

In addition, HAM/TSP patients have been demonstrated that the forkhead box P3 (FoxP3), which is critical for the function of regulatory T cells, was decreased in CD4^+^CD25^+^ T cells and regulatory function of the CD4^+^CD25^+^ T cells was also inhibited [47]. Based on these observations, we examined the expressions of FoxP3 and CTLA-4 in peripheral blood CD4^+^CD25^+^ T cells of HAM/TSP patients compared to NDs, ACs and patients with the other chronic virus infection and/or neuroinflammatory diseases. As previously reported [47], HAM/TSP patients showed decreased expressions of FoxP3 and CTLA-4 in CD4^+^CD25^+^ T cells of the peripheral blood compared to NDs and subjects with MS and PML (Fig 4C and 4D, respectively). These results strongly supported the previous finding that CD4^+^CD25^+^ T cells would be functionally dysregulated in HAM/TSP patients and also suggested that CD4^+^CD25^+^ T cells of HAM/TSP patients might be functionally different from those of subjects with the other neuroinflammatory diseases.

Our results demonstrated that CD4^+^CD25^+^ T cells were highly elevated in the CSF of HAM/TSP patients while memory Tfh cells were decreased. Moreover, comparison of CD4^+^ T cell subsets in neuroinflammatory diseases demonstrated that balances of memory Tfh cells and CD4^+^CD25^+^ T cells were different in each group although there were increases of B cells and ASCs in CSF across all groups.

### Correlation of CD4^+^ T cell subsets with ASCs in CSF

**Fig 5:**
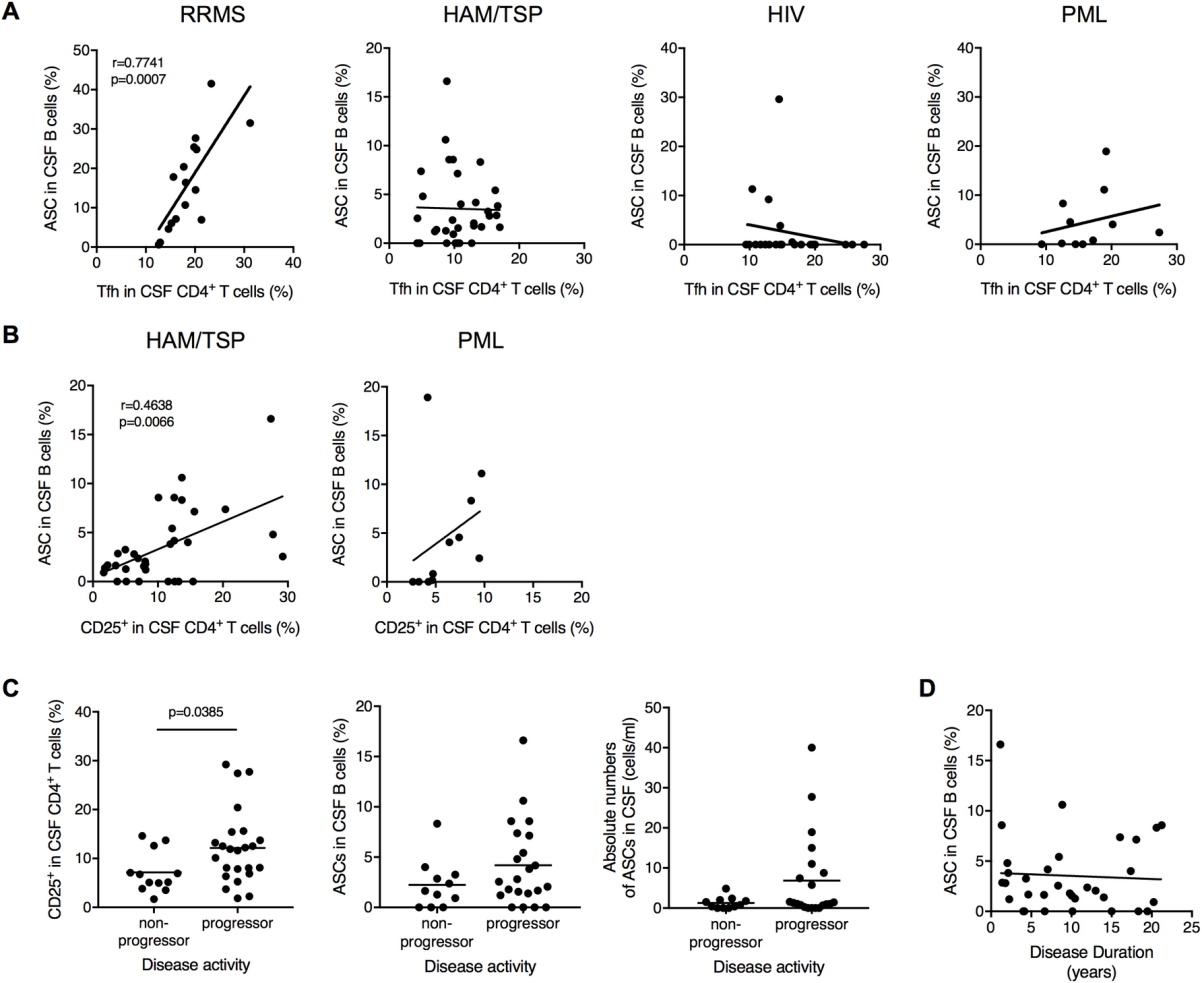
Correlation between ASC and CD4^+^ T cell subsets. (A) Correlation of ASCs with memory Tfh cells in CSF of patients with MS, HAM/TSP, HIV and PML using Spearman’s rank correlation test. (B) Correlation of ASCs with CD4^+^CD25^+^ T cells in CSF of patients with HAM/TSP and PML using Spearman’s rank correlation test. (C) Comparison of frequency of CD4^+^CD25^+^ T cells and ASCs in CSF of HAM/TSP patients between non-progressors and progressors using Mann-Whitney Test. The horizontal line represents the mean. (D) Correlation of ASCs in CSF B cells with disease duration in HAM/TSP patients (n = 32) using Spearman’s rank correlation test.

Given the characteristic features of CD4^+^ T cell subsets in CSF of HAM/TSP patients, we asked whether these CD4^+^ T cell subsets contribute to B cell regulation. To clarify the involvement of CD4^+^ T cells with B cell help in the CSF, the correlation of ASCs with memory Tfh cells and CD4^+^CD25^+^ T cells was analyzed in each group of HAM/TSP, RRMS, HIV and PML. While the frequency of memory Tfh cells was were significantly correlated with that of ASCs in the CSF of RRMS patients, surprisingly, there was no correlation between memory Tfh cells and ASCs in the CSF of subjects with HAM/TSP, HIV and PML (Fig 5A). By contrast, HAM/TSP patients showed significant correlation of CD4^+^CD25^+^ T cells with ASCs in the CSF (Fig 5B). Although PML patients had an increase of CD4^+^CD25^+^ T cells in the CSF compared to NDs (Fig 4A), PML patients did not show any correlation of CD4^+^CD25^+^ T cells and ASCs in the CSF (Fig 5B). These results strongly demonstrate that different subsets of CD4^+^ T cells, CD4^+^CD25^+^ T cells and memory Tfh cells, are involved in the increase of ASCs in CSF of HAM/TSP and RRMS patients, respectively.

Moreover, we compared CD4^+^CD25^+^ T cells and ASCs in the CSF with disease activity of HAM/TSP patients. When HAM/TSP patients were subdivided into two types, non-progressive and progressive types, characterized by disease course, progressive type of HAM/TSP patients showed significantly higher frequency of CD4^+^CD25^+^ T cells in the CSF compared to non-progressors (Fig 5C). However, both frequency and absolute number of ASCs in CSF B cells did not show any significant differences between these types of HAM/TSP patients (Fig 5C). In addition, there was no significant correlation of ASCs in CSF B cells with disease duration in HAM/TSP patients (Fig 5D). These results suggested that ASCs would not be directly involved in the disease activity of HAM/TSP patients but present for long periods in the CSF of HAM/TSP patients.

### Increased IL-21 expression in CD4^+^CD25^+^ T cells of HAM/TSP patients

**Fig 6:**
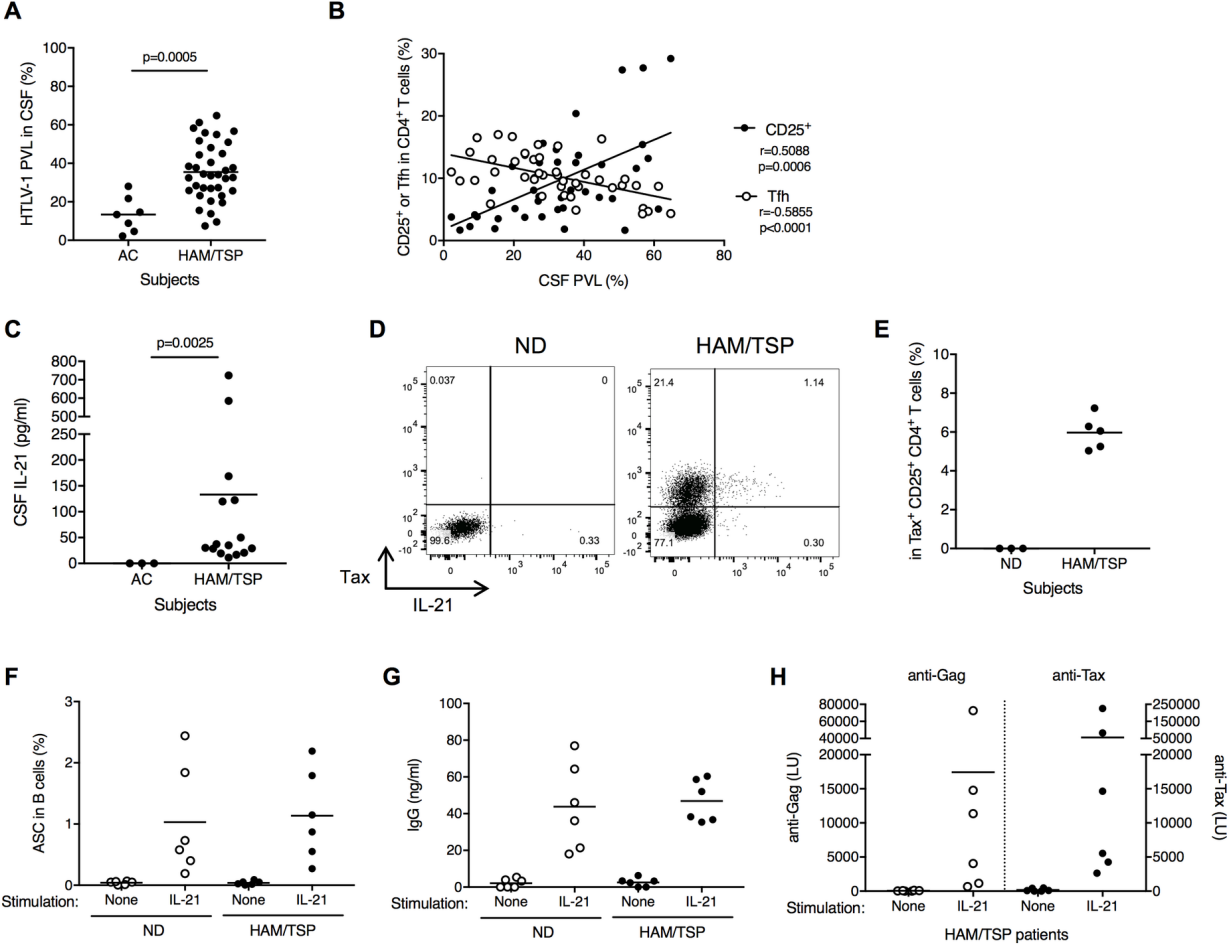
Involvement of CD4^+^CD25^+^ T cells with B cell help in CSF of HAM/TSP patients. (A) Comparison of HTLV-1 PVL in CSF of ACs (n = 7) and HAM/TSP patients (n = 36) using Mann-Whitney Test. (B) Correlation of HTLV-1 PVL with CD4^+^CD25^+^ T cells and memory Tfh cells in HTLV-1-infected subjects using Spearman’s rank correlation test. (C) Comparison of IL-21 in CSF of HAM/TSP patients and ACs using unpaired t test. (D) Representative dot plots of IL-21 and Tax staining in CD4^+^CD25^+^ T cells of a ND and a HAM/TSP patient after culture for 24 hours without any exogenous stimulation. (E) Detection of IL-21 in Tax-expressing CD4^+^CD25^+^ T cells of HAM/TSP patients after culture for 24 hours without any exogenous stimulation. (F) Generation of ASCs subsets in B cells cultured with and without rhIL-21. The data were obtained from cultured B cells of NDs and HAM/TSP patients (n = 6). The horizontal line represents the mean. (G) Detection of human IgG in the B cell culture supernatants of NDs and HAM/TSP patients. (H) Detection of antibodies for HTLV-1 Gag and Tax in the B cell culture supernatants of HAM/TSP patients.

Of the groups of patients with neuroinflammatory diseases assessed, only HAM/TSP patients showed an involvement of CD4^+^CD25^+^ T cells with an increase of ASCs in the CSF. To determine the role of HTLV-1 infection within these CD4^+^ T cell subsets in the CSF of HAM/TSP patients, we examined HTLV-1 PVL in the CSF lymphocytes of HTLV-1-infected subjects. HTLV-1 was detected in the CSF lymphocytes of all HTLV-1 subjects, and HTLV-1 PVL was significantly higher in HAM/TSP patients compared to ACs (Fig 6A). Further analysis revealed that CSF PVL significantly correlated with CD4^+^CD25^+^ T cells in the CSF of HTLV-1-infected subjects, but negatively correlated with memory Tfh cells in the CSF (Fig 6B). These results demonstrated that an increase of HTLV-1-infected cells is associated with an increase of CD4^+^CD25^+^ T cells and a decrease of memory Tfh cells in the CSF of HTLV-1 infected subjects, suggesting that CD4^+^CD25^+^ T cells with HTLV-1 infection could be involved with B cell help in HAM/TSP.

Tfh cells have been reported to express high levels of IL-21 which is a member of the common γ chain family of cytokines and promotes B-cell growth, differentiation, and class-switching [32, 39]. Since HTLV-1 Tax has been reported to transactivate IL-21 in human T cells [41], it is of interest whether or not IL-21 is involved with B cell help in HAM/TSP patients. We examined IL-21 in CSF of HAM/TSP patients and ACs. While IL-21 was undetectable in the CSF of ACs, high levels of IL-21 were detected in the CSF of HAM/TSP patients (Fig 6C). This suggests that the increase of IL-21 might be related to the increase of CD4^+^CD25^+^ T cells in the CSF of HAM/TSP patients as memory Tfh cells were decreased. To confirm IL-21 production in CD4^+^CD25^+^ T cells of HAM/TSP patients, we examined IL-21 expression in combination with HTLV-1 Tax expression in PBMCs of NDs and HAM/TSP patients. Fig 6D shows representative dot plots of HTLV-1 Tax and IL-21 expression in CD4^+^CD25^+^ T cells of a ND and a HAM/TSP patient after PBMC culture for 24 hours without any exogenous stimulation. IL-21 was detected in Tax-expressing CD4^+^CD25^+^ T cells of a HAM/TSP patient whereas ND did not show any IL-21 and Tax expression after culture (Fig 6D). Group analysis of five HAM/TSP patients demonstrated that Tax-expressing CD4^+^CD25^+^ T cells expressed IL-21 (Fig 6E). In addition, when B cells isolated from PBMC of NDs and HAM/TSP patients and stimulated with rhIL-21 for 7days, the frequency of ASCs, as well as IgG concentration, was significantly increased in rhIL-21-stimulated B cell cultures from both NDs and HAM/TSP patients (Fig 6F and 6G). Moreover, HTLV-1 Gag- and Tax-specific antibodies were also detected in the rhIL-21-stimulated B cell culture supernatants from HAM/TSP patients (Fig 6H). These results suggest that the increased IL-21 expression might be partly derived by HTLV-1 Tax expression in CD4^+^CD25^+^ T cells, and that it may serve to accelerate B cell function in HAM/TSP.

## Discussion

Regulation of the local immune response is crucial in protecting the CNS from viral infection and immunopathologically mediated tissue damage. Although robust humoral immune responses, including OCBs specific to viral antigens, have been reported in the CSF of patients with virus-associated neuroinflammatory diseases, little is known about the CNS microenvironment related to this increased humoral immune response in disease and healthy controls.

Multicolor flow cytometric analysis demonstrates that B cell/monocyte ratio and ASCs were increased in the CSF of HAM/TSP patients as well as RRMS patients, while ASCs were undetectable in CSF of NDs. Consistent with previous study [48], B cells were rarely detected in ND CSF, and when present, were in very low numbers in our study. Our results also strongly support recent studies in MS that in the B cell lineage, memory B cells and plasma blasts are predominantly detected in the CSF of MS patients [49]. Memory B cells can proliferate and rapidly differentiate into ASCs at much lower thresholds for activation than naïve B cells. Ig production is maintained by ASCs including proliferating “plasmablasts” and “plasma cells” that can be short or long-lived [50]. Therefore, it has been suggested that increased ASCs may be a marker for immune activation against a target, such as virus or host antigen, which is involved in disease development in neuroinflammatory disorders. In situ histopathological studies in spinal cord of HAM/TSP patients demonstrated that T cells including both CD4^+^ and CD8^+^ T cells were detected depending on the duration of illness whereas B cells were only rarely observed [6]. We here demonstrate an accumulation of B cells and elevated ASCs that may contribute to antibody production in the CSF of HAM/TSP patients. If CSF reflects the intrathecal inflammatory process in the CNS, increases of B cells and ASCs in the CSF might be an indicator of disease development in HAM/TSP. Importantly, ASCs were not detected in the CSF of ACs and a majority of HIV-infected subjects, although B cell phenotyping was able to be analyzed in only a small number of subjects with ACs. These results suggested that accumulation and differentiation of B cells might be well regulated in subjects with chronic virus infection but no neurologic diseases. Moreover, it is of interest that ASCs were also not detected in a subset of PPMS patients, while RRMS patients showed high frequency of ASCs in the CSF B cells. However, B cell is also important immune regulator for disease development of PPMS, since B cell depleting monoclonal antibody, Ocrelizumab, is the first drug ever to show efficacy in slowing the disease progression in a phase 3 clinical trial with PPMS patients [51]. Comparison of larger studies from MS patients would be required for confirmation of B cell phenotyping in CSF.

Antibody responses against HTLV-1 antigens including Gag and Tax were detected in CSF of all HAM/TSP patients tested. Interestingly, CSF/serum antibody index of anti-Gag and anti-Tax were significantly elevated in HAM/TSP patients while ACs had a significantly lower CSF/serum antibody index of all HTLV-1 antigens; this suggests that antibody responses for HTLV-1 Gag and Tax might be generated in HAM/TSP patients according to increases of viral expression or immune activation while antibody responses against these HTLV-1 antigens might be well controlled in the CSF of ACs. Moreover, increased ASCs in CSF of HAM/TSP patients significantly correlated with CSF HTLV-1 Gag-specific antibody production which was not observed in ACs. It is of interest that HAM/TSP patients have also been reported to develop autoantibodies to neurons that cross-reacted with HTLV-1 Gag and Tax [17, 18], suggesting that increased humoral immune responses including cross- or self-reactive antibodies to CNS antigens for HTLV-1 might alter the risk of CNS inflammation or autoimmune disease. Since HTLV-1-specific antibody responses and ASCs were stably detected in the CSF of HAM/TSP patients, persistent localization of ASCs may be associated with long-term stability of Ig production and OCB detection in the CSF of HAM/TSP patients. Intriguingly, antibody response for HTLV-1 Env was lower in the CSF of HAM/TSP patients compared to antibody responses for HTLV-1 Gag and Tax. Since neutralizing and antibody-dependent cellular cytotoxicity-inducing activity of antibodies against HTLV-1 Env gp46 have been reported to prevent viral infection *in vitro* [52], less robust antibody response for HTLV-1 Env may fail to control viral infection in the CNS of HAM/TSP patients. Therefore, it is important to identify target antigens of immune cells and antibodies for understanding disease development and therapeutic approach in chronic viral infection and neuroinflammatory diseases.

To maintain memory B cells and generate ASCs, a subset of CD4^+^ T cells called Tfh cells is required. Recently, it has been addressed that CXCR5^+^CD4^+^ T cells are detected in organs that are affected by autoimmune disorders, such as systemic lupus erythematosus and Sjogren’s syndrome, suggesting that aberrant Tfh cells may induce autoimmunity [53]. Recent reports in chronic HIV infection demonstrated that Tfh cells are expanded, but impair B cell help and harbor high amounts of viral DNA [53–55]. It has been demonstrated that precise control of Tfh cell number is important to produce optimally affinity-matured antibody responses that are devoid of self-reactivity [56]. In the current study, we demonstrate that subjects with chronic virus infection and/or neuroinflammatory diseases lost the balance of memory Tfh cell frequencies in CSF compared to CSF of NDs in which a certain frequency of memory Tfh cells were stably maintained. HAM/TSP patients showed a decrease of memory Tfh cells in the CSF. Importantly, memory Tfh cells were decreased in the CSF of ACs which did not show any intrathecal antibody synthesis for HTLV-1 or accumulation of CD4^+^CD25^+^ T cells in the CSF. Therefore, memory Tfh cell might be inhibited in the CSF of subjects with chronic HTLV-1 infection to prevent excess B cell responses but excessive accumulation and/or activation of CD4^+^ T cells might promote B cell development in HAM/TSP patients. Tfh cell responses have been reported to be regulated by various mechanisms, such as follicular regulatory CD4^+^ T cells in a Bcl6-dependent manner and CD8^+^ regulatory T cells in IL-15 dependent manner [56]. Further studies will be required to understand the regulation of memory Tfh cells associated with chronic virus infection in the CNS.

Tfh cells have also been reported to express high levels of IL-21 and promote B cell growth, differentiation and class switching [32]. It has been reported that IL-21 mRNA was elevated in peripheral blood CD4^+^ T cells of MS patients and IL-21 expressing CD4^+^ T cells were detected in MS lesions [36, 37], suggesting that a CD4^+^ T cell subset, memory Tfh cells, might be involved in B cell help through IL-21 in MS patients. While MS patients showed a significant correlation of ASCs with memory Tfh cells in the CSF, there was no association between ASCs and memory Tfh cells in the CSF of HAM/TSP patients. Interestingly, CD4^+^CD25^+^ T cells were significantly correlated with the frequency of ASCs as well as HTLV-1 PVL in the CSF of HAM/TSP patients, suggesting that chronic viral activation could induce continuous differentiation of memory B cells into ASCs and Ig production. Moreover, HAM/TSP patients showed an increase of IL-21 level in the CSF even though there was the decreased memory Tfh cells. Since ACs did not show any accumulation of CD4^+^CD25^+^ T cells and increased IL-21 in the CSF, this suggests that the increased IL-21 in HAM/TSP patients might be derived from CD4^+^CD25^+^ T cells in the CNS. IL-21 is a member of the common γ chain family of cytokine that also includes IL-2, IL-7, IL-9 and IL-15, and promotes B-cell growth, differentiation, and class-switching [39, 57]. After culture for 24 hours without any stimulation, IL-21 expression was detected in Tax-expressing CD4^+^CD25^+^ T cells of HAM/TSP patients. HTLV-1 Tax has been shown *in vitro* to induce the expression of IL-2 and IL-15 [40, 42]. Increased expression of these cytokines has been shown to dysregulate T-cell activation and proliferation that may contribute to CNS inflammation in HAM/TSP patients [58]. Although Tax has been reported to trans-activate IL-21 and its receptor (IL-21R) genes in human T cells [41], little is known about involvement of IL-21 in HAM/TSP patients. Since IL-2 and IL-15 have been also shown to be associated with B cell function, such as proliferation and Ig secretion [59], increased expression of these cytokines might accelerate B cell function in HAM/TSP patients. Alternatively, Tfh cell independent induction of B cell function might cause impaired B cell responses and generation of antigen-specific antibodies with low specificity and function. Therefore, adequate and appropriate Tfh cells for B cell help would be required for control of viral infection in the CNS. Larger systematic studies of virus-associated neurologic diseases including the functional differences of CD4^+^ T cell subsets will further improve our knowledge of the B cell/T cell immune regulation in the CNS associated with chronic viral infections.

Lastly, comparison of CSF immune phenotyping highlights that B cell/T cell interactions may be involved in the development and maturation of B cells in the CNS of neuroinflammatory diseases. Although ASCs were detected in high frequencies of patients with MS, HAM/TSP and PML, balances of CD4^+^ T cell subsets, memory Tfh cells and CD4^+^CD25^+^ T cells, were different in each group. Therefore, characterization of CSF immune responses that are associated with a neuroinflammatory milieu may provide evidence for a pathogenic “signature” of an immunopathogenic process in virus-associated neurologic diseases.

## Materials and methods

### Subjects

A total of 71 HAM/TSP patients and 12 HTLV-1-positive ACs were evaluated in this report according to established criteria [60]. To characterize patient’s disease onset based on clinical and motor outcomes, we used the Osame Motor Disability Score. Onset of disease was defined as rapidly progressive if the OMDS score increased by >3 grades since clinical onset of HAM/TSP [61]. HAM/TSP patients were further categorized as either progressors or non-progressors based on clinical status at the time of the lumbar puncture analysis if their symptoms were changing or stable, respectively. Subsets of HAM/TSP patients were used for specific studies. For detection of HTLV-1-specific antibodies, serum and CSF samples were obtained from a total of fifty subjects, including HAM/TSP patients (n = 44) and HTLV-1-positive ACs (n = 4). For flow cytometric analysis, whole blood was obtained from a total 170 subjects, including ACs, patients with HAM/TSP, MS including RRMS and PPMS [62], PML [63], HIV-infected subjects adequately treated with antiretroviral drugs and without neurological disease and NDs (Table 1). Of 170 subjects, CSF samples were obtained from a total 149 subjects (Table 1). PBMCs were isolated by Ficoll-Hypaque (Lonza) centrifugation, and were cryopreserved in liquid nitrogen until use. CSF samples were obtained by lumber puncture and the cells were collected by centrifugation of CSF samples.

### Ethics statement

The study was reviewed and approved by the National Institute of Neurological Disorders and Stroke Institutional Review Board. All samples used in this study were collected from the subject followed at the National Institute of Neurologic Disorders and Stroke under protocols # 98-N-0047, 89-N-0045, 13-N-0017, 13-N-0149. Prior to study inclusion, written informed consent was obtained from the subject in accordance with the Declaration of Helsinki.

### Luciferase Immunoprecipitation Systems (LIPS)

The LIPS assay was performed as previously described [11]. Each mammalian expression vector with the HTLV-1 gene (HTLV-1 Gag, Env and Tax/pRen2) was transfected into 293T/17 cell line (ATCC) using X-tremeGENE 9 DNA transfection reagent (Roche Diagnostics) [11]. Serum, CSF samples or B cell culture supernatants were diluted to 1:100. Each HTLV-1-specific antibody index was calculated as ratio of CSF immunoreactivity (LU)/serum immunoreactivity (LU).

### Flow cytometry

For analysis of peripheral blood lymphocyte and CSF lymphocyte populations, EDTA-treated whole blood or CSF cells were stained with CD3, CD4, CD8, CD14, CD19, CD25, CD27, CD45, CD45RA, CXCR5, IgD (all from BD Biosciences) and PD-1 (BioLegend). Since B cells (CD45^+^CD3^-^CD19^+^) were rarely detected in CSF, CSF samples from a total 93 subjects were used for B cell subset analysis (Table 2). For staining of FoxP3 and CTLA-4, EDTA-treated whole blood were stained with antibodies for surface markers. After fixed and permeabilized with Fixation/Permeabilization buffer (eBiosciences), the cells were stained with antibodies for FoxP3 (eBiosciences) and CD152 (CTLA-4; BD Biosciences).

For detection of IL-21 production, PBMCs of NDs or HAM/TSP patients were suspended in RPMI media (RPMI1640 supplemented with 10% heat-inactivated fetal bovine serum, 100U/ml of penicillin, 100μg/ml of streptomycin sulfate and 2mM L-glutamine) for 24 hours and incubated with GoldiPlug (BD Biosciences) for the last 5 hours in 5% CO_2_ incubator at 37°C. After the culture without any stimulator, PBMCs were surface-stained with specific antibodies. After fixation and permeabilization with Fixation/Permeabilization solution (BD Biosciences), the cells were intracellularly stained with anti-human IL-21 (BD Biosciences) and anti-Tax (Lt-4) antibodies. All flow cytometric analysis was performed using a LSR II (BD Biosciences). The data were analyzed using FlowJo 10.2 software (FlowJo LLC).

### HTLV-1 PVL

HTLV-1 PVL was measured using droplet digital PCR (Bio-Rad) as previously described [64]. DNA was extracted from the PBMC and CSF cell pellets using a DNeasy Blood and Tissue kit (Qiagen) according to the manufacturer’s instructions. Primers and probes specific for HTLV-1 *tax* and human ribonuclease P protein subunit 30 (RPP30) was used. The duplex PCR amplification was performed in this sealed 96-well plate using a GeneAmp 9700 thermocycler (Applied Biosystems). Following PCR amplification, the 96-well plate was transferred to a QX100 droplet reader (Bio-Rad). For PVL calculation, QuantaSoft software version 1.3.2.0 (Bio-Rad) was used to quantify the copies/μl of each queried target per well. All samples were tested in duplicate, unless otherwise specified, and PVL is reported as the average of the two measurements.

### ELISA

IL-21 were detected in serum and CSF samples of HAM/TSP patients and ACs using Human Legend Max Human IL-21 ELISA kit (BioLegend) according to the manufacturer’s instructions.

### B cell differentiation and antibody production

B cells were isolated from PBMCs of NDs and HAM/TSP patients using B cell isolation kit II (Miltenyi Biotec). The isolated B cells were cultured at 2x10^4^ cells/well in 96 U-bottom microplates in RPMI media with or without 10ng/ml of recombinant human IL-21 (Cell Signaling Technology). After the culture for 7 days, the cells were surface-stained with specific antibodies and analyzed using a LSR II (BD Biosciences). Human IgG and HTLV-1-specific antibodies were measured in the culture supernatants using Human IgG ELISA Quantitation Set (Bethyl Laboratories) and LIPS assay, respectively.

### Statistics

The Mann-Whitney Test was used to compare: anti-HTLV-1 antibodies, PVL and IL-21 in CSF between ACs and HAM/TSP patients, CSF lymphocytes in HAM/STP patients by disease activity. Paired T Test was used to compare: anti-HTLV-1 antibody indexes in each HTLV-1-infected subject. The Kruskal-Wallis test with Dunn’s test for multiple testing was used to compare: frequency or ratio of CSF lymphocytes between the different patient groups. Fisher’s exact test was used to compare frequency of CSF ASCs detection in each patient’s group with NDs. Spearman’s rank correlation test was used to compare: the CSF/serum anti-HTLV-1 antibody index, HAM/TSP disease duration, CD4^+^ T cell subsets with ASCs in CSF, and between CD4^+^ T cell subsets and PVL in CSF. All statistical analysis was performed using Prism (GraphPad software).
